# The effect of Mg location on Co-Mg-Ru/γ-Al_2_O_3_ Fischer–Tropsch catalysts

**DOI:** 10.1098/rsta.2015.0087

**Published:** 2016-02-28

**Authors:** James R. Gallagher, Paul Boldrin, Gary B. Combes, Don Ozkaya, Dan I. Enache, Peter R. Ellis, Gordon Kelly, John B. Claridge, Matthew J. Rosseinsky

**Affiliations:** 1 Department of Chemistry, University of Liverpool, Liverpool L69 7ZD, UK; 2 Johnson Matthey PLC, PO Box 1, Belasis Avenue, Billingham TS23 1LB, UK; 3 Johnson Matthey PLC, Blount’s Court, Sonning Common, Reading RG4 9NH, UK

**Keywords:** Fischer–Tropsch, catalysis, cobalt, alumina, XRD

## Abstract

The effectiveness of Mg as a promoter of Co-Ru/γ-Al_2_O_3_ Fischer–Tropsch catalysts depends on how and when the Mg is added. When the Mg is impregnated into the support before the Co and Ru addition, some Mg is incorporated into the support in the form of Mg_*x*_Al_2_O_3+*x*_ if the material is calcined at 550°C or 800°C after the impregnation, while the remainder is present as amorphous MgO/MgCO_3_ phases. After subsequent Co-Ru impregnation Mg_*x*_Co_3−*x*_O_4_ is formed which decomposes on reduction, leading to Co(0) particles intimately mixed with Mg, as shown by high-resolution transmission electron microscopy. The process of impregnating Co into an Mg-modified support results in dissolution of the amorphous Mg, and it is this Mg which is then incorporated into Mg_*x*_Co_3−*x*_O_4_. Acid washing or higher temperature calcination after Mg impregnation can remove most of this amorphous Mg, resulting in lower values of *x* in Mg_*x*_Co_3−*x*_O_4_. Catalytic testing of these materials reveals that Mg incorporation into the Co oxide phase is severely detrimental to the site-time yield, while Mg incorporation into the support may provide some enhancement of activity at high temperature.

## 1. Introduction

Fischer–Tropsch synthesis (FTS) is an increasingly important industrial reaction for the production of hydrocarbons from a mixture of carbon monoxide and hydrogen, known as syngas. FTS can be used to convert cheap gas sources, such as shale gas, to exploit stranded gas reserves, to prevent gas flaring during oil extraction or to convert feedstocks such as coal and biomass to higher value liquid hydrocarbons [1–5]. The resulting hydrocarbons after work-up comprise a mixture of liquid hydrocarbons with low sulfur and aromatic content, therefore giving access to clean-burning, synthetic fuels. Hence, FTS is a key step in gas-to-liquid, coal-to-liquid and biomass-to-liquid technologies.

Cobalt catalysts for FTS generally consist of the active metal dispersed as an oxide over an oxidic support with various quantities of metal oxide or platinum group metal promoters to improve the reducibility of the active phase. As it is widely accepted that FTS proceeds over Co metal sites [6], prior to use, catalysts are usually activated with H_2_ *in situ* to reduce the Co oxides to Co metal.

Magnesium has been used numerous times as an additive to FTS catalysts. Indeed, some early work used Mg as a promoter in a Co/MgO/ThO_2_/kieselguhr catalyst [7]. Most studies have used Mg to improve the mechanical strength and hence attrition resistance by the formation of spinels [8]. However, a study by Zhang *et al.* [9] found that Mg loaded at 0.8 wt% on γ-Al_2_O_3_ suppressed the formation of difficult to reduce Co-surface phases. The effect was thought to be due to the formation of MgAl_2_O_4_ spinel on the surface of the γ-Al_2_O_3_, which reduced the contact between Co and γ-Al_2_O_3_ and therefore the amount of cobalt aluminate spinel, which is not FT-active. Hence, Mg at low loadings could increase reducibility and activity. The same study found that more than 1 wt% MgO decreased the reducibility due to the formation of an MgO–CoO solid solution, which required high-temperature reduction. Chernavskii *et al.* [10] also found an increase in reducibility when Mg was loaded at 0.2 wt% on an γ-Al_2_O_3_ support. A study by Niemelä *et al.* [11] found that MgO-promoted Co/SiO_2_-supported catalysts reduced the X-ray diffraction (XRD) particle size of Co metal particles from 14 nm to around 10 nm and decreased reducibility presumably due to the smaller particle size. It should be noted that Co/SiO_2_ catalysts tend to be more reducible than Co/γ-Al_2_O_3_ catalysts and therefore a decrease in reducibility of MgO-promoted SiO_2_ may be expected. The study by Niemelä also found that activity was higher when Mg was impregnated after Co onto the support rather than before. A more recent study by Holmen’s group observed a decrease in activity for Mg-promoted Co/γ-Al_2_O_3_ catalysts which could not be explained by particle size or dispersion effects [12]. The authors suggest that Mg could have a poisoning effect similar to that of alkali metals. Work by our group using proxy-based accelerated discovery methods has shown that the order of addition of the Mg has a large effect on the size of the Co particles formed and also on the activity and stability of the catalysts [13]. Mg in general reduces particle size and activity, while adding Mg before the other elements and calcining at 550°C produces catalysts with high stability.

In this work, we aim to identify the origin of the effects on particle size, activity and stability. We conduct detailed characterization of Mg-promoted catalysts, and a series of experiments aimed at controlling the location of Mg in the catalysts to better understand the effects.

## 2. Experimental set-up

Catalysts were synthesized by an incipient wetness impregnation process. The support, γ-Al_2_O_3_ (Sasol; Puralox TH100/150, 145 m^2^ g^−1^), was dried at 120°C overnight. The impregnation was carried out in plastic bags (Tesco; zipper seal sandwich bags, 18×18 cm). The impregnation was a multi-step process, the first step being the impregnation of magnesium nitrate hexahydrate (Fluka; greater than or equal to 99%) followed by heat treatment at either 550°C or 800°C, then the impregnation of the cobalt nitrate hexahydrate (Acros; 99%) and ruthenium (III) nitrosyl nitrate (Alfa Aesar; min 31.3% Ru), followed by a final heat treatment at 250°C or 400°C. To the γ-Al_2_O_3_ was added the appropriate amount of hot aqueous nitrate solution (*ca* 60°C) to achieve the desired loading. The impregnated solid was then kneaded in the plastic bag until homogeneous. For thermal treatments, the damp solid was spread out on a stainless steel tray and treated in static air in a muffle furnace. After thermal treatment, subsequent impregnations were carried out using the same procedure. The volume used for the initial impregnation was 1 ml g^−1^ for unmodified Puralox TH100/150, and the volume required was recalculated after each impregnation.

Some of the Mg-modified supports were subjected to an acid wash. Two different solutions with different pH were used to wash the Mg/γ-Al_2_O_3_ materials—acetic acid (1 mol dm^−3^, pH 2.4) and an acetic acid–ammonium acetate buffer (0.5 mol dm^−3^, pH 4.7). Acetic acid was chosen as it has a similar pH to Co(NO_3_)_2_ (ranges from approx. 3 at 1 M concentration to approx. 2 at 4 M) and the buffer solution was chosen to determine whether a milder treatment could produce the same results. Additionally, residual species left after the washes could be removed by appropriate heat treatments, unlike for instance acetic acid–sodium acetate buffers. The washing procedure was thus:

To each Mg/γ-Al_2_O_3_ sample (1 g) contained in a centrifuge tube (50 ml) was added the weak acid solution (40 ml) followed by shaking for 30 s. Tubes were then centrifuged for 30 min at 4000 r.p.m. before removing the supernatant with a pipette. In some cases, the Mg and Al content of the supernatant was analysed by inductively coupled plasma (ICP) analysis. The wet solid was washed with water (40 ml) via shaking by hand for 30 s before returning to the centrifuge for 30 min at 4000 r.p.m. The supernatant was removed by pipette before drying the wet solid for 18 h at 80°C. The dry solid was calcined at 550°C for 2 h in static air to remove residual acetate or ammonium ions.

XRD data on samples from Boldrin *et al.* [13] (supports and catalysts) and on the catalysts in the acid washing study described above were collected on a Panalytical X-Pert diffractometer fitted with a X-Celerator detector and a spinner stage set up in Bragg–Brentano geometry. X-rays were produced with a Co anode filtered by Ge giving monochromatic K_*α*1_ radiation (1.7890 Å). Variable divergence slits were used with an illuminated length of 15 mm. The measuring time for XRD scans in the 2*θ* range 18°–82° was 1 h. Samples were spiked with KCl in order to more accurately calculate the lattice parameters of the Co_3_O_4_ phase. Two methods were used to extract quantitative information from XRD patterns: empirical peak fitting and Le Bail whole pattern fitting. Empirical peak fitting was used to calculate particle size from the Scherrer equation while Le Bail fitting was used to calculate the lattice parameters of Co_3_O_4_ phases. TOPAS 4.2 was used to refine the patterns of the Co_3_O_4_/γ-Al_2_O_3_ samples. In addition to the background polynomial, the lattice parameters and effective particle size parameters were refined, although determination of instrumental broadening was not performed so these particle size measurements are not discussed as they would lead to a false impression of their accuracy. Where particle size measurements are quoted, these are calculated from single peaks using the Scherrer equation.

In addition to this, the acid-washed (AW) supports were characterized using the high-throughput XRD technique described by Boldrin *et al.* [13]. Samples were placed in well-plates, adhesive tape was stuck over the tops of all wells and the plates were upturned to stick the sample to the tape to ensure that the sample height was the same across all wells. High-throughput XRD was performed in reflection mode on a Panalytical X-Pert Pro diffractometer fitted with an XYZ stage using Co K_*α*1_/*K*_*α*2_ (ratio=2) radiation, Fe filter, 5 mm mask and automatic divergence slits with an illuminated length of 3 mm. The Soller slits used were 0.02° on the incident side and 0.04° on the reflection side. The measuring time for an XRD scan in the 2*θ* range 38°–57° was 13 min.

Fourier transform infrared (FTIR) spectra were recorded for Mg-modified γ-Al_2_O_3_. Spectra were collected from 600 to 4000 cm^−1^ with a resolution of 4 cm^−1^ using a Perkin Elmer Spectrum 100 spectrometer fitted with the Spectrum 100 Universal Diamond/ZnSe ATR.

Temperature-programmed reduction (TPR) was carried out on an Altamira AMI-200 unit. Samples (70 mg) were purged with flowing argon at 30 ml min^−1^ before the gas was switched to 10% H_2_/Ar at 30 ml min^−1^. Samples were ramped from room temperature to 1100°C at 10°C min^−1^, holding at 1100°C for 10 min. Five injections were performed at the end of the experiment, which allowed the peak area to be related to H_2_ consumption.

Thermogravimetric analysis (TGA) scans were carried out on a TA Q5000IR thermal analyser using 5% H_2_/N_2_ as the sample gas flowing at 25 ml min^−1^. Samples (*ca* 5 mg) were placed in aluminium pans. The dynamic high-resolution mode was used with a ramp rate of 50°C min^−1^, sensitivity of 1.0 and a resolution of 4.0. Samples were heated from room temperature to 600°C, where the temperature was held for 5 min. The maximum temperature was limited to 600°C due to the use of aluminium pans.

CO_2_ temperature-programmed desorption (TPD) was carried out on an Altamira AMI-200 unit. Samples (250 mg) were first heated to 250°C at 10°C min^−1^ and purged with flowing He at 30 ml min^−1^ for 10 min before being cooled to 35°C at 10°C min^−1^. Subsequently, the gas was switched to 10% CO_2_/He at 30 ml min^−1^ for 1 h at 35°C followed by 30 ml min^−1^ He for 30 min. Finally, the sample was heated to 800°C at 10°C min^−1^ in He flowing at 10 ml min^−1^, holding at 800°C for 10 min. Five injections were performed at the end of the experiment, which allowed the peak area to be related to CO_2_ desorption.

Co surface areas were determined by H_2_ chemisorption at 150°C in a Micromeritics ASAP 2020C by extrapolating the total gas uptakes in the H_2_ adsorption isotherms at zero pressure. Prior to adsorption, the samples (*ca* 0.5 g) were pre-treated in flowing He at 120°C for 1 h. Afterwards, the samples were reduced *in situ* by flowing pure H_2_ and raising the temperature to 425°C and maintaining this temperature for 6 h. After reduction, the samples were degassed, the temperature lowered to 150°C and H_2_ dosed over the sample in the pressure range of 100–760 mm Hg.

Transmission electron microscopy was performed to calculate Co particle size distributions and to produce elemental maps. Samples were first reduced and passivated. Powder samples were embedded in resin, cured, microtomed and placed on holey carbon-coated Cu grids. The samples were examined in the probe-corrected JEOL-2100F transmission electron microscope (TEM) at the University of Birmingham, UK, using the scanning transmission mode with high-angle annular dark-field and bright-field detectors and a voltage of 200 kV. Parallel electron energy loss spectroscopy (EELS) using a GATAN Enfina spectrometer was used to investigate the composition.

ICP analysis was performed to determine the compositions of solid samples. Following complete dissolution of the samples using microwave digestion in HCl, analysis was performed on a Perkin Elmer Optima ICP-OES

ICP analysis was performed to determine the amount of Mg and Al ions removed from Mg-modified γ-Al_2_O_3_ solids by washing with acetic acid or ammonium acetate on a Ciros CCD optical emission spectrometer.

The FTS testing was carried out in a stainless steel isothermal fixed bed six-way micro-reactor connected to an online Varian CP-3800 gas chromatograph with three detectors. A Valco-valve was used to select between the gas streams for online analysis. Catalysts were reduced at 425°C for 9 h in pure H_2_ prior to testing. After reduction, the reactor was cooled to 160°C and H_2_ was replaced with syngas (H_2_:CO:Ar=2:1:0.1) and pressure was raised to 20 bar. Conversion was measured by the consumption of the syngas mix (CO+H_2_) relative to Ar. Wax and water were collected from the gas–liquid separators and analysed using an offline gas chromatogram with a flame ionization detector on a SimDist column in order to calculate the chain growth probability, *α*, from the C_22_–C_40_ fraction.

## 3. Results and discussion

In our previous work [13], we described the high-throughput discovery, scale-up and testing of a series of Co-Ru catalysts supported on Mg-modified γ-Al_2_O_3_. The Mg was found to have several benefits: reducing the Co particle size, improving the Co surface area and increasing the stability of the catalysts during FTS. There were also detrimental effects on the reducibility of the catalysts and the initial activity. In order to investigate the origin of these effects, detailed characterization was carried out on both the initial catalysts and the modified supports.

XRD analysis of the calcined catalysts (electronic supplementary material, figure S1) showed the presence of two phases, one assigned to the γ-Al_2_O_3_ support and one assigned to Co_3_O_4_. Le Bail refinements were undertaken with peaks inserted empirically for the alumina phase due to the difficulty in assigning a structural model to this phase [14,15], with the results shown in table 1. The lattice parameter of the Co_3_O_4_ phase for the 0% Mg sample was slightly smaller than expected for bulk Co_3_O_4_. This slight contraction of the unit cell could be the result of the small size (14.7 nm) of the Co_3_O_4_ particles causing deviations from bulk properties, for instance, due to the presence of vacancies or other crystal imperfections [17]. As the Mg loading increased, the Co_3_O_4_ lattice parameter increased. By comparison with Mg_0.93_Co_2.07_O_4_, which is reported to have a lattice parameter of 8.1390 Å[16], and considering that Mg^2+^ has a larger ionic radius than Co^2+^ (86 pm versus 79 pm in octahedral sites [18]) this can be attributed to incorporation of Mg into the Co_3_O_4_ lattice forming a solid solution with general formula Mg_*x*_Co_3−*x*_O_4_. This implies that, prior to Co(NO_3_)_2_ incipient wetness impregnation, there are Mg species which are sufficiently reactive to form a mixed oxide with Co ions. By comparison with the literature data on the lattice parameter of Mg_*x*_Co_3−*x*_O_4_ compounds [17], an estimate of the amount of Mg in the Mg_*x*_Co_3−*x*_O_4_ spinel relative to the total amount of Mg can be calculated. For the 6% Mg sample, the composition of the spinel was Mg_0.76_Co_2.24_O_4_, which means that 62% of the total magnesium in the sample is incorporated in this phase. The particle sizes calculated from the broadening of the Co_3_O_4_ peaks indicate that catalysts containing Mg had smaller Co_3_O_4_ particles than the catalyst without Mg, which agrees with our previous work [13].

**Table 1.** XRF and XRD data for catalysts from Boldrin *et al.* [13]. LP, lattice parameter; PS, particle size. Amount of Mg incorporated into Mg_*x*_Co_3−*x*_O_4_ spinel was calculated by comparison with literature values reported by Krezhov & Konstantinov [16]. This can then be used to estimate the mass of the Mg in the Mg_*x*_Co_3−*x*_O_4_ and, by subtraction from the total mass of Mg, the amount unaccounted for, described as ‘other Mg’ in the final column.

	XRF				XRD					
nominal Mg (wt%)	Mg (wt%)	Al (wt%)	Co (wt%)	Ru (wt%)	Co_3_O_4_ LP (Å)	Co_3_O_4_ PS (nm)	Al_2_O_3_ LP (Å)	*x* in Mg_*x*_Co_3−*x*_O_4_	wt% of Mg in Mg_*x*_Co_3−*x*_O_4_	wt% of other Mg
0	0.00	36.83	18.23	0.08	8.0770 (3)	14.7	7.9010 (15)	0.00	n.a.	n.a.
0.5	0.26	36.86	17.18	0.06	8.0807 (4)	11.5	7.9072 (16)	0.09	0.21	0.05
3	2.06	33.28	19.58	0.06	8.0937 (4)	11.3	7.9187 (15)	0.36	1.09	0.97
6	4.13	31.07	18.26	0.05	8.1190 (4)	11.2	7.9330 (14)	0.76	2.56	1.57

Although a Le Bail refinement could not be performed on the γ-Al_2_O_3_ phase, information relating to the phase can be gathered by simply looking at the shifts in the peaks. Although somewhat crude, by assuming that γ-Al_2_O_3_ has the defect spinel structure, a lattice parameter was calculated by measuring the peak position of a single reflection at approximately 53.6° 2*θ* (table 1). It can be seen that, as the Mg loading increased, the γ-Al_2_O_3_ peaks shifted to the lower 2*θ* position, indicating an expansion of the interplanar distances and hence an increase in the defect spinel lattice parameter. This indicates that Mg was incorporating into the γ-Al_2_O_3_ structure to form a phase with general formula Mg_*y*_Al_2_O_3+*y*_ since the defect spinel structure for γ-Al_2_O_3_ has a lattice parameter of 7.9 Åand the lattice parameter for the spinel MgAl_2_O_4_ is 8.0824 Å(reference pattern: ICDD 021-1152). The modified supports were also characterized by XRD to examine the crystalline phases present before Co(NO_3_)_2_ impregnation. This analysis confirmed that the shift in lattice parameter of the γ-Al_2_O_3_ phase observed in the Co-loaded catalysts was primarily due to Mg incorporation (electronic supplementary material, figure S2). All XRD patterns only contained peaks due to the Mg_*y*_Al_2_O_3+*y*_ phase except for the sample with 6% Mg calcined at 550°C, which also contained peaks due to MgO. The emergence of the MgO peaks only at a loading of 6% Mg indicates that below 6% Mg the MgO particles were too small or insufficiently crystalline to be detected by XRD under the experimental set-up. Because peaks due to MgO were seen in this sample before Co(NO_3_)_2_ impregnation and calcination but not seen after, this indicates that Mg_*x*_Co_3−*x*_O_4_ formation in the catalyst is probably due to a dissolution or solid-state reaction causing Mg ions to incorporate into the Co_3_O_4_ structure. The lattice parameter for the γ-Al_2_O_3_ phase is similar before and after Co(NO_3_)_2_ impregnation, suggesting that the Mg in Mg_*x*_Co_3−*x*_O_4_ does not come from Mg_*y*_Al_2_O_3+*y*_ and is therefore likely to be from an amorphous or highly dispersed Mg-containing phase.

It is evident from the XRD data that, in the pre-activated catalyst, Mg is incorporated into the Co_3_O_4_ *and* the γ-Al_2_O_3_ structures. From the measurement of *x* in Mg_*x*_Co_3−*x*_O_4_, it is possible to calculate the proportion of Mg which is present in the Mg_*x*_Co_3−*x*_O_4_ phase, and, by subtraction, the remaining Mg which is not accounted for by this (given as ‘other Mg’ in table 1). If all the remaining Mg was incorporated in the γ-Al_2_O_3_ phase to form Mg_*y*_Al_2_O_3+*y*_, we would expect the lattice parameter of this phase to increase proportionally to the ‘other Mg’ content, and this does somewhat seem to be the case, with a linear regression giving an *r*^2^-value of 0.966, although with only four points this is inconclusive. The absence of any peaks due to MgO or other Mg-containing phase (e.g. MgCO_3_) indicates that any Mg which was not incorporated into Mg_*x*_Co_3−*x*_O_4_ or Mg_*y*_Al_2_O_3+*y*_ was highly dispersed or poorly crystalline. The proportion of the total Mg which is incorporated into the Mg_*x*_Co_3−*x*_O_4_ phase increases with increasing Mg loading.

Mg/γ-Al_2_O_3_ samples were calcined at a higher temperature of 800°C to provide insight into the behaviour of the samples calcined at the lower temperature of 550°C. The XRD patterns showed a single Mg_*y*_Al_2_O_3+*y*_ phase for all compositions and, again, an increase in the lattice parameter with Mg loading was observed (electronic supplementary material, figure S2). However, for these samples, the increase in the lattice parameter was greater than that at the lower calcination temperature. This indicates that more Mg was incorporated into the γ-Al_2_O_3_ structure, presumably due to the higher temperature facilitating more diffusion into the bulk. As the lattice parameter increased more at 800°C, this implies that after calcination at 550°C there are Mg species which are XRD-silent and not incorporated into the Mg_*y*_Al_2_O_3+*y*_ phase. As this available Mg was not seen by XRD for samples other than 6% Mg calcined at 550°C this means that it is either poorly crystalline or highly dispersed, perhaps as a monolayer as suggested previously [19]. It is these XRD-silent Mg species which are the likely origin of the Mg in Mg_*x*_Co_3−*x*_O_4_.

**Figure 1.**
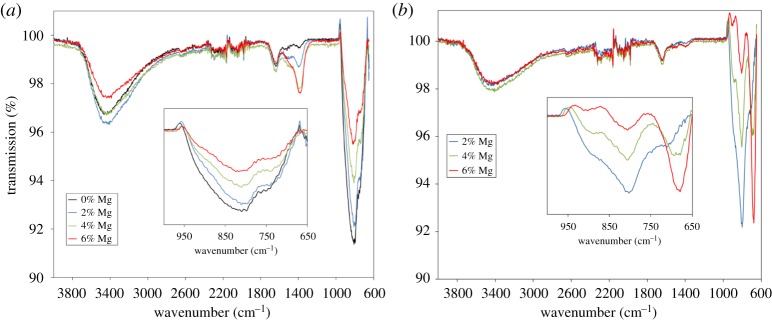
(*a*) FTIR spectra for Mg/γ-Al_2_O_3_ modified supports with different Mg loadings calcined at 550°C. Unmodified γ-Al_2_O_3_ is shown for comparison. (*b*) FTIR spectra for Mg/Al_2_O_3_ modified supports with different Mg loadings calcined at 800°C. Insets show close-ups of the 650–1000 cm^−1^ region, where modes due to Al–O bonds are found.

FTIR spectroscopy on the modified supports (figure 1) can give information about both the XRD-silent Mg species and the Mg_*y*_Al_2_O_3+*y*_ phase. As expected, every sample had major bands due to O−H (*ca* 3500 cm^−1^), which is mainly due to adsorbed water in the pore network but may also have a contribution from hydroxide species. The presence of a peak at *ca* 1385 cm^−1^ in the samples calcined at 550°C is indicative of carbonate ions. The intensity of this peak increased with Mg loading, indicating that MgCO_3_ was present. This peak is absent in the samples calcined at 800°C. CHN analysis confirms the presence of carbon in all the modified supports calcined at 550°C, and the two highest loadings of Mg in the supports calcined at 800°C (electronic supplementary material, figure S3). The amount of carbon increases with Mg loading, reinforcing the hypothesis that the peaks in the FTIR are due to MgCO_3_. CHN analyses conducted over time show an increasing carbon content with time since synthesis (electronic supplementary material, figure S4), suggesting that initially the XRD-silent Mg species are amorphous oxides, which convert to carbonates over time in air.

Examination of the low-energy region of the FTIR spectrum reveals peaks typically due to metal–oxygen bonds and gives information about the Mg_*y*_Al_2_O_3+*y*_ phase. For the samples calcined at 550°C, there is a peak at *ca* 824 cm^−1^ which is attributed to tetrahedral Al−O bonds [20]. As the Mg loading increased the intensity of this band decreased. A similar trend was observed for the samples calcined at 800°C but to a greater extent (figure 1*b*). Furthermore, for the samples calcined at 800°C, an additional band at *ca* 683 cm^−1^, attributed to octahedral Al−O bonds [21], increased in intensity as Mg loading increased. These changes in intensity with Mg loading indicate that, as the Mg loading increases, there are fewer Al^3+^ ions in tetrahedral sites and more in octahedral sites. In a normal spinel such as MgAl_2_O_4_, the divalent cation resides in tetrahedral sites and the trivalent cation is in octahedral sites. Although the structure of γ-Al_2_O_3_ is similar to spinel, it is often reported as having many defects, non-spinel sites and with a proportion of the Al^3+^ cations in tetrahedral sites rather than octahedral [15,21]. The FTIR data suggest that, as the Mg loading and calcination temperature was increased, the Al^3+^ cations’ occupation of crystallographic sites resembles more closely that of bulk MgAl_2_O_4_ spinel. Presumably, as the Mg loading increases, more Mg^2+^ is incorporated into the tetrahedral sites of the γ-Al_2_O_3_, which results in fewer Al^3+^ cations in the tetrahedral sites and more in the octahedral sites. This move towards a spinel-type Mg_*y*_Al_2_O_3+*y*_ structure is consistent with the XRD data.

Further information about the nature of the supports can be deduced from CO_2_ TPD (electronic supplementary material, figure S5). The unmodified support possesses a single CO_2_ desorption peak at around 100°C, indicating the presence of a weakly basic site, as is expected from γ-Al_2_O_3_ [15,19]. This is also the case with all the modified supports calcined at 800°C, as well as the ones calcined at 550°C with an Mg loading of less than 2%. This suggests that incorporation of Mg into the γ-Al_2_O_3_ crystal structure does not increase the basicity of the support’s surface. The total amount of CO_2_ desorbed does not change with Mg loading in these samples, which reinforces this hypothesis. However, modified supports with Mg loadings of 2% and above calcined at 550°C also possessed a more strongly basic site reflected in an additional desorption peak at 300°C. This is likely to be from the XRD-silent Mg species identified previously, especially as, in these samples, the total amount of CO_2_ does increase with Mg loading.

Comparing the total CO_2_ desorption in the TPD with the amount of Mg present in each phase in the final catalyst, we can see that the correlation is better with the amount of Mg in the Mg_*x*_Co_3−*x*_O_4_ phase than with the amount of ‘other’ Mg. Because the total amount of CO_2_ desorbed is mainly influenced by the XRD-silent Mg, this suggests that the XRD-silent Mg is ending up in the Mg_*x*_Co_3−*x*_O_4_ phase rather than as ‘other’ Mg in the final catalyst, so the ‘other’ Mg is almost entirely the Mg_*y*_Al_2_O_3+*y*_ phase (rather than being a combination of Mg in Mg_*y*_Al_2_O_3+*y*_ and XRD-silent Mg species). This is in agreement with the XRD data, which show a good correlation between the Mg_*y*_Al_2_O_3+*y*_ lattice parameter and the ‘other Mg’ content, and gives an indication that the cobalt impregnation solution is dissolving the majority of the amorphous Mg, which is then incorporated into the Mg_*x*_Co_3−*x*_O_4_ phase.

The evidence detailed above strongly suggests that the modification of γ-Al_2_O_3_ with Mg has two effects: (i) the creation of an Mg_*y*_Al_2_O_3+*y*_ phase that is intermediate between γ-Al_2_O_3_ and MgAl_2_O_4_ in crystal structure, which has a similar basicity to the unmodified support and is unchanged by the impregnation of cobalt, and (ii) the deposition of highly basic XRD-silent MgO or MgCO_3_ species which are later incorporated into the cobalt oxide spinel forming Mg_*x*_Co_3−*x*_O_4_. We hypothesized that it is the MgO/MgCO_3_ species that is dissolved by the acidic Co(NO_3_)_2_ impregnation solution and thus forms the Mg_*x*_Co_3−*x*_O_4_ phase. We therefore attempted to remove this second species by acid washing the modified supports before Co(NO_3_)_2_ impregnation, allowing us to separately examine the two effects. The washing solutions chosen were acetic acid (pH 2.4), which has a similar pH to concentrated cobalt nitrate solutions (pH 2–3 in the range of 3–4 M concentrations), and ammonium acetate buffer (pH 4.7) to assess if a milder treatment could achieve the same results.

**Figure 2.**
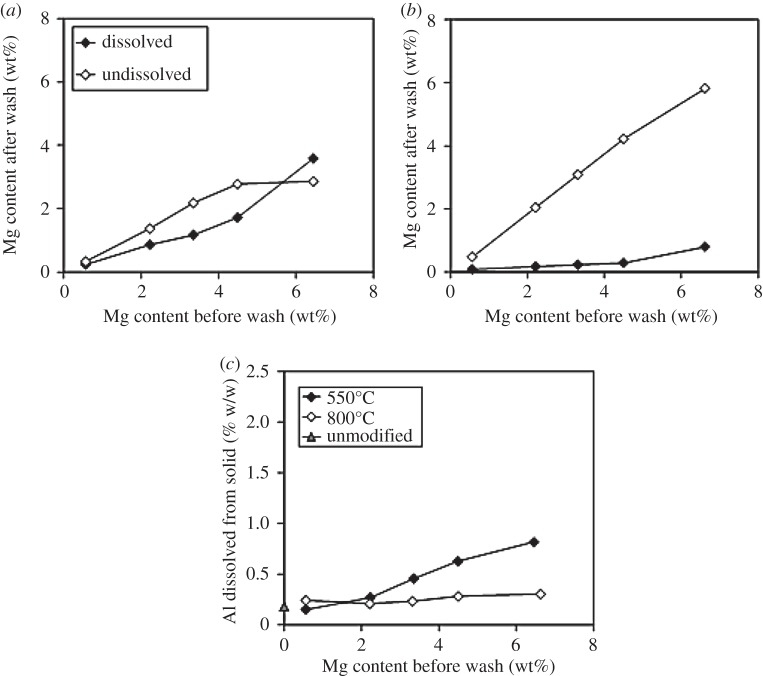
(*a*,*b*) Amount of Mg dissolved or not dissolved from Mg/γ-Al_2_O_3_ modified supports calcined after Mg addition at either (*a*) 550°C or (*b*) 800°C washed with ammonium acetate buffer. (*c*) Amount of Al dissolved from Mg/γ-Al_2_O_3_ modified supports calcined after Mg addition at either 550°C or 800°C washed with ammonium acetate buffer. Washed, unmodified γ-Al_2_O_3_ is shown for comparison.

ICP analysis of the supernatant allowed us to characterize the amount of Mg removed during washing, while the amount left behind is calculated by subtraction from the ICP results of the materials before washing. The results are shown in figure 2*a*,*b* (buffer wash) and the electronic supplementary material, figure S6 (acetic acid wash). Washing with either acetic acid (pH 2.4) or ammonium acetate buffer (pH 4.7) resulted in almost identical amounts of Mg being dissolved from the sample for both the supports calcined at 550°C and 800°C, indicating that the two washing solutions were equally effective in dissolving Mg. As would be expected, much more Mg was dissolved from samples calcined at 550°C than from those calcined at 800°C. Interestingly, some Al was also dissolved by both ammonium acetate buffer and acetic acid (figure 2*c* and electronic supplementary material, figure S7, respectively), including, in the unmodified support, raising the prospect that some Al may also be incorporated into the Co_3_O_4_ spinel, an effect which to our knowledge has not been discussed previously in the literature on γ-Al_2_O_3_-supported catalysts. The amount of Al dissolved increases with Mg loading, suggesting that Al in mixed Mg-Al species is more easily dissolved. Less Al is dissolved by the higher pH buffer.

**Figure 3.**
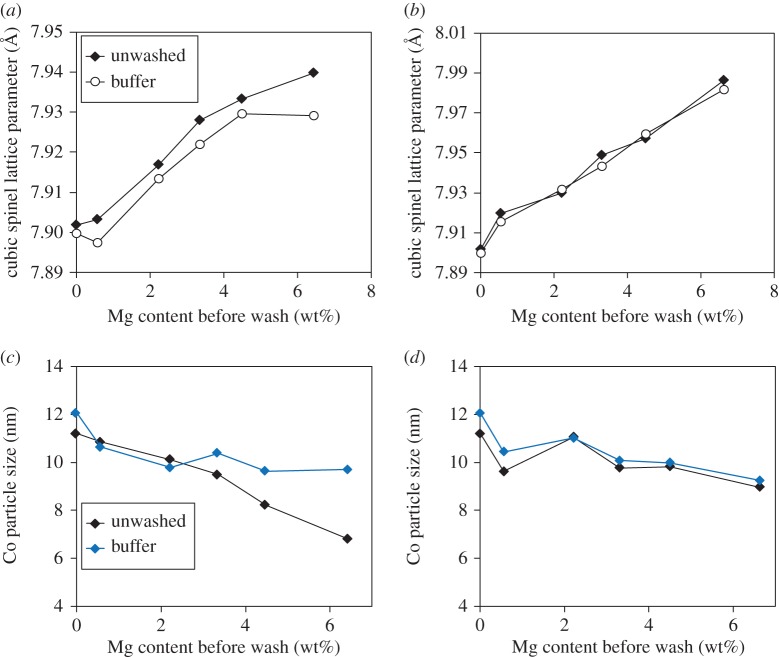
(*a*,*b*) Variation with Mg loading of lattice parameter of Mg/γ-Al_2_O_3_ modified supports washed with either acetic acid or ammonium acetate buffer for samples calcined after Mg addition at either (*a*) 550°C or (*b*) 800°C. Lattice parameters were calculated assuming a cubic spinel structure for γ-Al_2_O_3_. Lattice parameters of unwashed samples are shown for comparison, (*c*,*d*) Co metal particle size calculated from high-throughput XRD [13] for catalysts synthesized using the supports from (*a*,*b*) calcined after Mg addition at either (*c*) 550°C or (*d*) 800°C. Data shown are the mean of two aliquots.

Comparison of the lattice parameters of the Mg_*y*_Al_2_O_3+*y*_ phase from XRD of the washed and unwashed samples (figure 3*a*,*b*) shows little change on washing for all samples, indicating that this phase is largely stable towards washing with acid. There is a small decrease in the lattice parameters for the samples calcined at 550°C, and this is reflective of the ICP results given above, which suggest that, although the majority of the dissolved Mg comes from highly dispersed or amorphous Mg-containing phases, some mixed Mg-Al species are also dissolved.

A series of Co-Ru catalysts were made using the washed and unwashed supports calcined at 550°C and 800°C, and the Co_3_O_4_ crystallite size was measured using XRD (figure 3*c*,*d*). In samples where the support has been calcined at 550°C, Mg loading still has an effect in reducing the particle size, but the effect is reduced compared with unwashed samples. In addition, higher loadings of Mg show a bigger difference in Co particle size between washed and unwashed samples, suggesting that a large part of the particle size reduction is due to the Mg species which are removed by washing. The washed and unwashed supports calcined at 800°C give very similar particle sizes to those seen in the washed samples calcined at 550°C, which again indicates that it is the free Mg which largely causes the decrease in particle size.

TGA in 5% H_2_ in N_2_ was carried out on these catalysts. Samples where the supports were calcined at 550°C show clearly that washing with either acid or buffer improves reducibility, as measured by the temperature maxima for the second reduction peak (Co(II)–Co(0); electronic supplementary material, figure S8). This is in line with the hypothesis that it is the free Mg which is detrimental to reducibility rather than total Mg.

Several samples washed with ammonium acetate buffer solution (henceforth AW) were scaled up for characterization and testing based on these results. Characterization of these samples is shown in table 2. Acetic acid samples were scaled up and characterized, but as there was no obvious difference between the acid- and buffer-washed samples these are not discussed further. Data from the acetic AW samples are shown in the electronic supplementary material, table S1.

**Table 2.** Summary of XRF, XRD, TPR and H_2_ chemisorption data for scaled up Co/Ru/Mg/γ-Al_2_O_3_ catalysts prepared with ammonium acetate buffer washes after Mg addition. Amount of Mg incorporated into Mg_*x*_Co_3−*x*_O_4_ spinel was calculated by comparison with the literature values reported by Krezhov & Konstantinov [16]. This can then be used to estimate the mass of the Mg in the Mg_*x*_Co_3−*x*_O_4_ and, by subtraction from the total mass of Mg, the amount unaccounted for.

				XRF			XRD					TPR		H_2_ chemisorption
sample	nominal Mg loading (wt%)	calcination *T* after Mg addition (°C)	wash	Mg (wt%)	Al (wt%)	Co (wt%)	Co_3_O_4_ lattice parameter (Å)	*x* in Mg_*x*_ Co_3−*x*_O_4_	Mg in Mg_*x*_Co_3−*x*_O_4_ (%)	other Mg (%)	Co_3_O_4_ PS (nm)	peak pos. (°C)	peak pos. (°C)	CoSA (m^2^/g_Co_)
AW1	0	n.a.	n.a.	0.00	36.57	17.08	8.0796 (3)	0.00	0	0	13.9	259	439	57.4
AW2	3	550	n.a.	2.64	34.89	17.37	8.0889 (4)	0.21	0.54	2.10	13.3	283	501	63.9
AW3	3	550	buff	1.94	35.08	17.08	8.0834 (3)	0.09	0.22	1.72	11.5	291	501	61.5
AW4	3	800	n.a.	2.64	35.11	17.21	8.0801 (3)	0.01	0.02	2.62	11.9	275	466	57.5
AW5	6	550	buff	2.56	34.84	16.95	8.0859 (4)	0.15	0.37	2.19	11.3	303	545	62.5
AW6	6	800	n.a.	5.18	33.09	17.25	8.0833 (3)	0.09	0.22	4.96	13.0	281	510	58.6

XRD analysis showed that the washed catalysts still contained some Mg_*x*_Co_3−*x*_O_4_, indicating that the washing is not fully effective in preventing this phase from forming. It is not clear why the impregnation solution is able to dissolve Mg from the washed supports, possibly because the impregnation solution is hot, or there is some other chemical effect than simply the pH. The washes were effective in reducing the Mg in Mg_*x*_Co_3−*x*_O_4_ by 60% in the 3% Mg samples, and by 80% in the 6% Mg samples. The washes also removed an appreciable fraction of the Mg not incorporated into Mg_*x*_Co_3−*x*_O_4_—around 17% in the 3% Mg samples and 33% in the 6% Mg samples. The calcination at 800°C was more effective at reducing the formation of Mg_*x*_Co_3−*x*_O_4_—in the 3% Mg sample less than 1% of the total Mg was found in the Mg_*x*_Co_3−*x*_O_4_ phase, while in the 6% Mg sample it was 4% of the total Mg.

The crystallite size of the Mg_*x*_Co_3−*x*_O_4_ spinel phase can be calculated from XRD, and it can be seen that acid washing has a strong effect in reducing the particle size. The reason for this is unclear, and it occurs even in samples without Mg (electronic supplementary material, table S1). The relative effects of the Mg in Mg_*x*_Co_3−*x*_O_4_ and Mg in Mg_*x*_Al_2_O_3+*x*_ on particle size are difficult to deconvolute, because they are heavily autocorrelated (i.e. samples which have more Mg in the Mg_*x*_Co_3−*x*_O_4_ phase also have more in the Mg_*x*_Al_2_O_3+*x*_ phase), and the calcination at 800°C which would provide a comparator also has a separate effect on particle size. In general, the results agree with earlier results that higher Mg contents reduce particle size.

**Figure 4.**
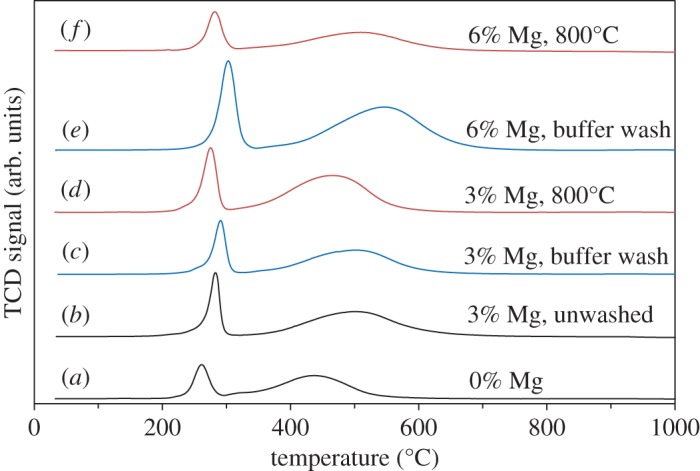
TPR data for Co/Ru/Mg/γ-Al_2_O_3_ catalysts prepared with acid washes after Mg addition. Traces are for samples (*a*) AW1, (*b*) AW2, (*c*) AW3, (*d*) AW4, (*e*) AW5 and (*f*) AW6. Black refers to catalyst prepared without acid wash; blue to buffer solution wash; and red to unwashed, calcined at 800°C after Mg addition.

The effect of the different Mg levels on the reducibility, as seen in the TPR, is somewhat clearer (figure 4). The washing lowers the reduction temperature for the samples with the highest Mg loading of 6%, and this manifests itself in both a lowering of the peak for the Co(II)–Co(0) reduction and a sharpening of the reduction peak, consistent with the hypothesis that it is the amount of Mg in Mg_*x*_Co_3−*x*_O_4_ rather than the total amount of Mg which determines the reduction temperature. Calcining the support at 800°C also lowers the reduction temperature relative to both washed and unwashed samples with the same initial Mg loading, and this also reinforces the hypothesis. This is complicated slightly by the 3% Mg samples calcined at 550°C, which show no change in peak position between washed and unwashed samples despite a large difference in the level of Mg in Mg_*x*_Co_3−*x*_O_4_. However, taking these results together with the results of TGA in 5% H_2_ carried out on the earlier samples (electronic supplementary material, figure S8), it becomes clear that it is the Mg in Mg_*x*_Co_3−*x*_O_4_ which is detrimental to reducibility.

The cobalt surface area measurements as determined by H_2_ chemisorption followed the same trend for the unwashed samples as the Mg-promoted samples scaled up previously but were slightly lower, which could be due to the higher calcination temperature. The Co surface area increased from 57.4 m^2^ g^−1^_Co_ for 0% Mg to 63.9 m^2^ g^−1^_Co_ for 3% Mg before decreasing to 59.2 m^2^ g^−1^_Co_ for 6% Mg. Again this is attributed to decreasing particle size coupled with decreasing reducibility as the Mg loading was *increased*.

AW2–AW6 were tested in the Fischer–Tropsch reaction to examine the effect of acid washing and high-temperature calcination prior to Co addition, along with a control sample, which was the 0% Mg sample discussed at the beginning of the Results section and in our previous work. To avoid confusion, we will refer to this sample as 0% Mg. The main difference between this sample and AW1 is that AW1 had a final heat treatment at 450°C rather than at 250°C, and a slightly lower Co surface area of 9.8 m^2^/g_cat_ compared with 11.2 m^2^/g_cat_. The testing procedure involved examining the activity and selectivity at 210°C, 230°C and 240°C, before returning to 210°C over a total of 245 h time online at testing temperature.

**Figure 5.**
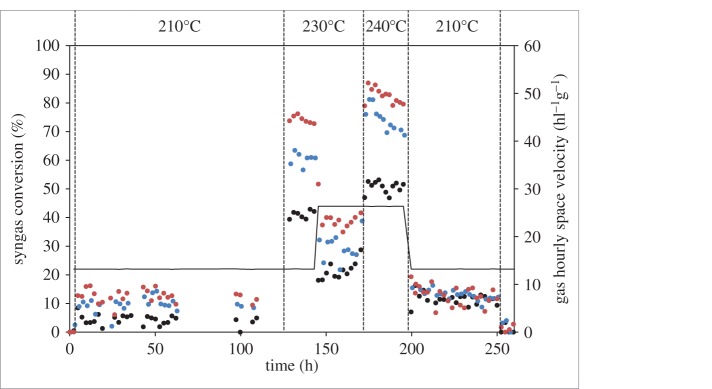
Syngas conversions (symbols) and gas hourly space velocity (line) over the time of the FTS tests for AW2 (black circles), AW3 (blue circles) and AW4 (red circles).

Figure 5 shows the testing data for three samples—AW2, AW3 and AW4—with these three samples all containing 3% Mg, either without (AW2) or with (AW3) an acid wash, or with a calcination of 800°C after Mg impregnation (AW4). It can be clearly seen that AW4 has the highest activity at all temperatures, followed by AW3, with AW2 having the lowest activity. This clearly shows the beneficial effect of acid washing and higher temperature calcinations on the activity. Plots of testing data for all samples are shown in the electronic supplementary material, figures S9 (activity) and S10 (selectivity).

The trends in activity at 210°C (shown in table 3) cannot be explained by the Co surface area data measured by chemisorption (table 2). For example, aside from the 0% Mg standard, the highest Co surface area was measured for AW2, but this had the lowest activity. Despite the correlation between the Mg content in Mg_*x*_Co_3−*x*_O_4_ and reducibility discussed above, it appears that the trends in activity cannot be explained simply by reducibility; for example, AW5 has a second reduction peak 35°C higher than any other catalyst but still has higher activity than AW2 or AW6.

**Table 3.** Fischer–Tropsch activity data for Co/Ru/Mg/γ-Al_2_O_3_ catalysts at 210°C. Stabilities are calculated from the change in activity or stability between the initial period of testing at 210°C and the final period at 210°C after the middle high-temperature period. Errors shown are standard deviations. Site-time yield (STY) calculated using Co surface area from *ex situ* H_2_ chemisorption. Chain growth probability, *α*, calculated from offline analysis of C_22_–C_40_ fractions. GHSV, gas hourly space velocity.

					initial selectivity			stability				
sample	initial interval (h)	final interval (h)	GHSV (l g^−1^ h^−1^)	initial syngas conversion (%)	CH_4_ (%)	C_2_–C_4_ (%)	C_5+_ (%)	syngas conversion (%)	CH_4_ (%)	C_5+_ (%)	*α*	STY (×10^−3^ s^−1^)
0% Mg	6–108	203–249	13.16	21.5±2.5	11.4±0.9	2.0±0.2	86.6±1.0	86±13	94±10	100±2	0.88	37
AW2	7–109	200–248	13.20	4.0±1.7	14.0±0.9	4.2±0.2	81.7±1.0	275±124	72±11	106±4	0.84	10
AW3	5–108	200–248	13.27	9.6±2.4	11.2±1.9	2.5±0.5	86.3±2.4	137±39	87±19	99±4	0.86	19
AW4	5–109	202–248	13.22	12.5±2.4	11.6±1.3	2.3±0.4	86.1±1.6	101±31	99±19	94±7	0.87	25
AW5	6–108	201–249	13.13	9.1±2.3	11.4±2.2	3.0±1.0	85.6±3.1	150±51	82±18	103±4	0.85	17
AW6	7–109	204–249	13.57	7.3±2.6	12.4±2.8	3.2±0.8	84.4±3.5	150±63	84±23	104±5	0.83	15

**Figure 6.**
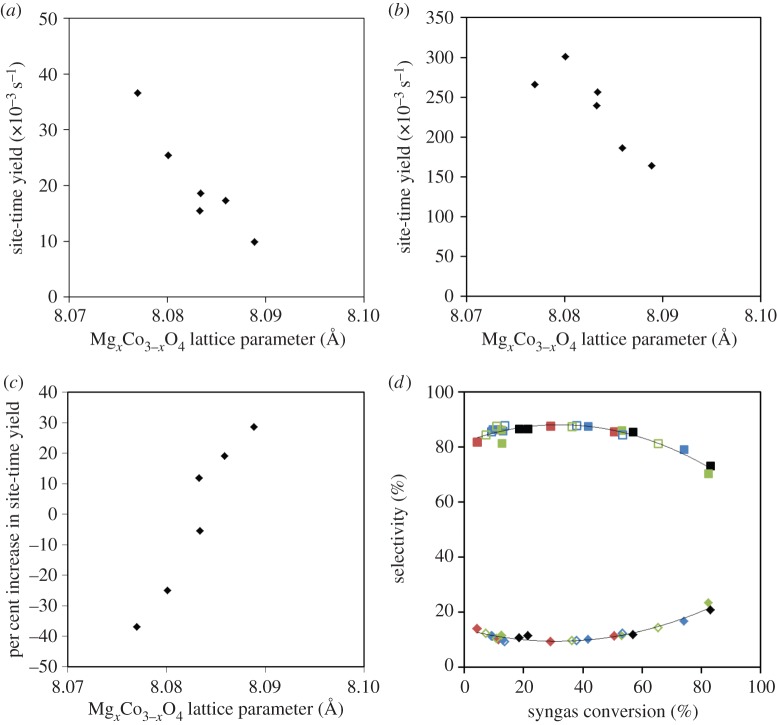
(*a*,*b*) Relationship between site-time yield and Mg_*x*_Co_3−*x*_O_4_ at (*a*) 210°C and (*b*) 240°C. (*c*) Relationship between percentage change in site-time yield comparing initial and final periods at 210°C. (*d*) Selectivities towards C_5+_ (squares) and CH_4_ (diamonds) for all samples at all temperatures. The standard is black; filled symbols have 3% Mg; unfilled symbols have 6% Mg; blue symbols are calcined at 550°C and washed; red symbols are calcined at 550°C and unwashed; green symbols are calcined at 800°C.

However, calculating the site-time yields (activity per H chemisorption site, measured *ex situ*) of each catalyst at 210°C and plotting them against the lattice parameter of the Mg_*x*_Co_3−*x*_O_4_ phase measured in the as-calcined state provides insight into the trends in activity just discussed. Figure 6*a* shows the site-time yields with respect to the lattice parameter for all six catalysts. It is clear that the level of Mg substitution into the Co_3_O_4_ spinel has a marked effect on the intrinsic activity of the catalysts—as the Mg_*x*_Co_3−*x*_O_4_ phase becomes more Mg-rich, the site-time yield decreases approximately linearly with increasing lattice parameter.

**Figure 7.**
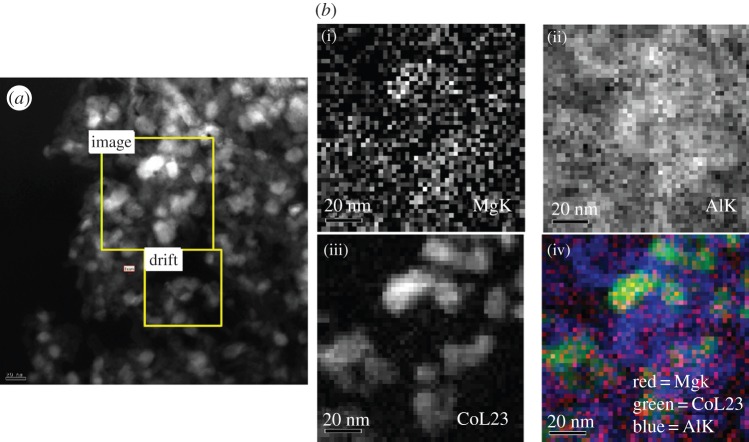
(*a*) High-resolution TEM micrograph of 3% Mg and (*b*) the same region measured using EELS to give elemental maps of the amount of (i) Mg, (ii) Al and (iii) Co, and (iv) superposition of all three elementalmaps. The brighter the pixel the higher the concentration of the element.

Because a single-mixed Co-Mg phase is formed after calcination, it is highly likely that, on reduction in the reactor, Mg which was mixed intimately with Co remains in close proximity to the reduced Co metal atoms. Hence the more Mg-rich the mixed spinel, the greater the amount of Mg in close proximity to the Co metal active sites in the activated catalyst. In order to investigate this, EELS was applied to a reduced and passivated 3% Mg catalyst (figure 7). The catalyst was reduced in pure H_2_ at 425°C for 9 h followed by passivation in 1% O_2_ in Ar at room temperature. The results indicate that Mg remains in close proximity to Co after reduction because the concentration distribution of Mg roughly maps that of Co. Presumably, on reduction, Co(III) and Co(II) ions are reduced to Co(0), destroying the Mg_*x*_Co_3−*x*_O_4_ structure but remaining in close proximity to the Mg ions which they were previously intimately mixed with.

Hence, it is proposed that Mg ions have a poisoning effect on the intrinsic activity of Co metal sites and the degree of poisoning depends on the concentration of Mg ions close to the Co sites. The decrease in activity with increasing Mg incorporation into Co_3_O_4_ appears not to be simply due to the total amount of Mg present in the sample, exemplified by the fact that AW6 has almost twice the total amount of Mg as AW2 but approximately 150% of the activity at 210°C.

Poisoning by Ca has been observed previously with the reduction in activity being attributed to electronic effects causing a decrease in surface hydrogen concentrations and increased CO adsorption and dissociation [22]. Another study investigating the poisoning effect of alkali and alkaline earth metals in the context of impurity elements in biomass-derived syngas implied that Mg had a poisoning effect but that it was less than for Ca. The authors suggested that a physical site-blocking effect could be the cause [23].

After approximately 65 h at high temperature, the catalysts were returned to 210°C. Interestingly, all the samples except the 0% Mg standard had higher activity after the high-temperature run than before, as shown in table 3 by stability values more than 100%. This suggests that, rather than deactivating the catalysts, the high-temperature period led to changes in the catalysts which actually *activated* them. The percentage increase in activity is strongly related to the level of Mg in the Mg_*x*_Co_3−*x*_O_4_ (figure 6*c*), suggesting that the higher temperatures are causing Mg to migrate away from Co, causing a reduction in the poisoning effect of Mg.

During the high-temperature phase of the test at 230°C and then 240°C, the site-time yields of all the catalysts except AW5 increase relative to the 0% Mg standard sample, to the extent that, by 240°C, AW4 has a higher site-time yield than the standard, while AW3 is the same within error (figure 6*b*). Again, the relative increase in site-time yield is somewhat correlated to the level of Mg in the Mg_*x*_Co_3−*x*_O_4_, although as the yields fall back below those of the standard when returning to 210°C, this effect cannot be entirely due to a high-temperature activation process and must be at least somewhat an intrinsic property of Mg promotion. Arrhenius plots for the 0% Mg standard, AW3 and AW4 (electronic supplementary material, figure S11) show no deviation from a linear trend at the highest temperature, which also indicates that there is no large change in the number of active sites. The fact that it is AW4, in which *x* in Mg_*x*_Co_3−*x*_O_4_ is only 0.01, which shows the largest increase in site-time yield with temperature raises the interesting prospect that the improved high-temperature performance is due to Mg incorporated into the alumina phase.

Looking at the selectivity of the samples (figure 6*d*), there is a consistent correlation between selectivity for all products and conversion among all samples at all temperatures, strongly suggesting that Mg in any form is not having a detectable influence on the selectivity or on the relative rates of possible competing reactions such as water gas shift.

## 4. Conclusion

The fact that magnesium is incorporated into the cobalt oxide spinel phase as well as the alumina phase despite the high-temperature treatment before impregnation has important implications for the use of structural promoters. We have shown that, while incorporation into the alumina phase may have beneficial effects on the high-temperature performance, incorporation into the cobalt phase has severely deleterious effects on the site-time yield, which may hold for other structural promoters. We have also shown that the impregnation solution has leaching effects greater than its pH implies, and also that the solution is potentially capable of dissolving measurable amounts of aluminium from the support, which presumably is also incorporated into the cobalt spinel phase, with unknown effects.

Quantification of the extent and effect of this doping of promoters and elements from the support into different structures of a catalyst is a difficult problem requiring detailed investigation with XRD, high-resolution TEM and elemental analysis, as well as a carefully designed experimental strategy, but should be a fruitful avenue of research in assessing new materials and reassessing older materials.

## Supplementary Material

Supplementary information for “The effect of Mg location on Co-Mg-Ru/?-Al2O3 Fischer-Tropsch catalysts”

## Data accessibility

The datasets supporting this article are available (doi:10.17638/datacat.liverpool.ac.uk/69).
