# Supplementary material for: The effect of Mg location on Co-Mg-Ru/γ-Al2O3 Fischer–Tropsch catalysts
Source: Philos Trans A Math Phys Eng Sci. 2016 Feb 28;374(2061):20150087. doi: 10.1098/rsta.2015.0087 (PMC4707690; doi:10.1098/rsta.2015.0087)
Supplement: Supplementary information for “The effect of Mg location on Co-Mg-Ru/?-Al2O3 Fischer-Tropsch catalysts” [file rsta20150087supp1.docx]

**Supplementary information for “The effect of Mg location on Co-Mg-Ru/γ-Al_2_O_3_ Fischer-Tropsch catalysts”**


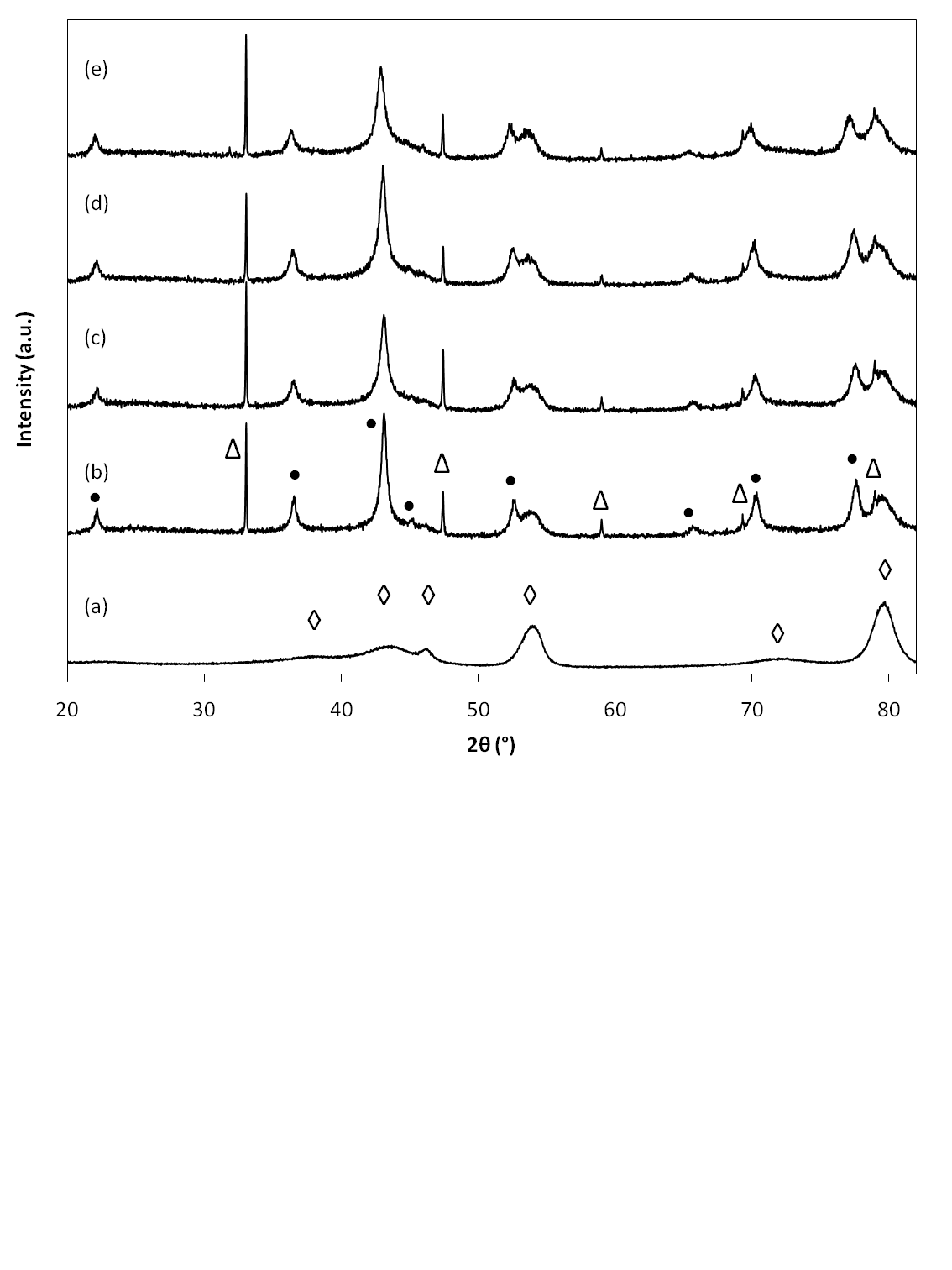


**Figure S1** XRD patterns of (a) unmodified Al_2_O_3_ support and; as-calcined catalysts (b) 0% Mg; (c) 0.5% Mg; (d) 3% Mg and (e) 6% Mg. Compositions as in Table 1. Symbols: ◊ Al_2_O_3_; • Mg_x_Co_3-x_O_4_; ∆ KCl internal standard.

**Figure S2** Variation with Mg loading of lattice parameter of Mg/Al_2_O_3_ modified supports calcined at either 550 °C or 800 °C. Lattice parameters were calculated assuming a cubic spinel structure for γ-Al_2_O_3_.

**Figure S3** Carbon content (wt%) from CHN micronalysis for Mg/γ-Al_2_O_3_ modified supports with different Mg loadings calcined at 550 °C or 800 °C.

**Figure S4** Carbon content (wt%) from CHN micronalysis as a function of time since synthesis for 3% Mg support calcined at either 550 or 800 °C.


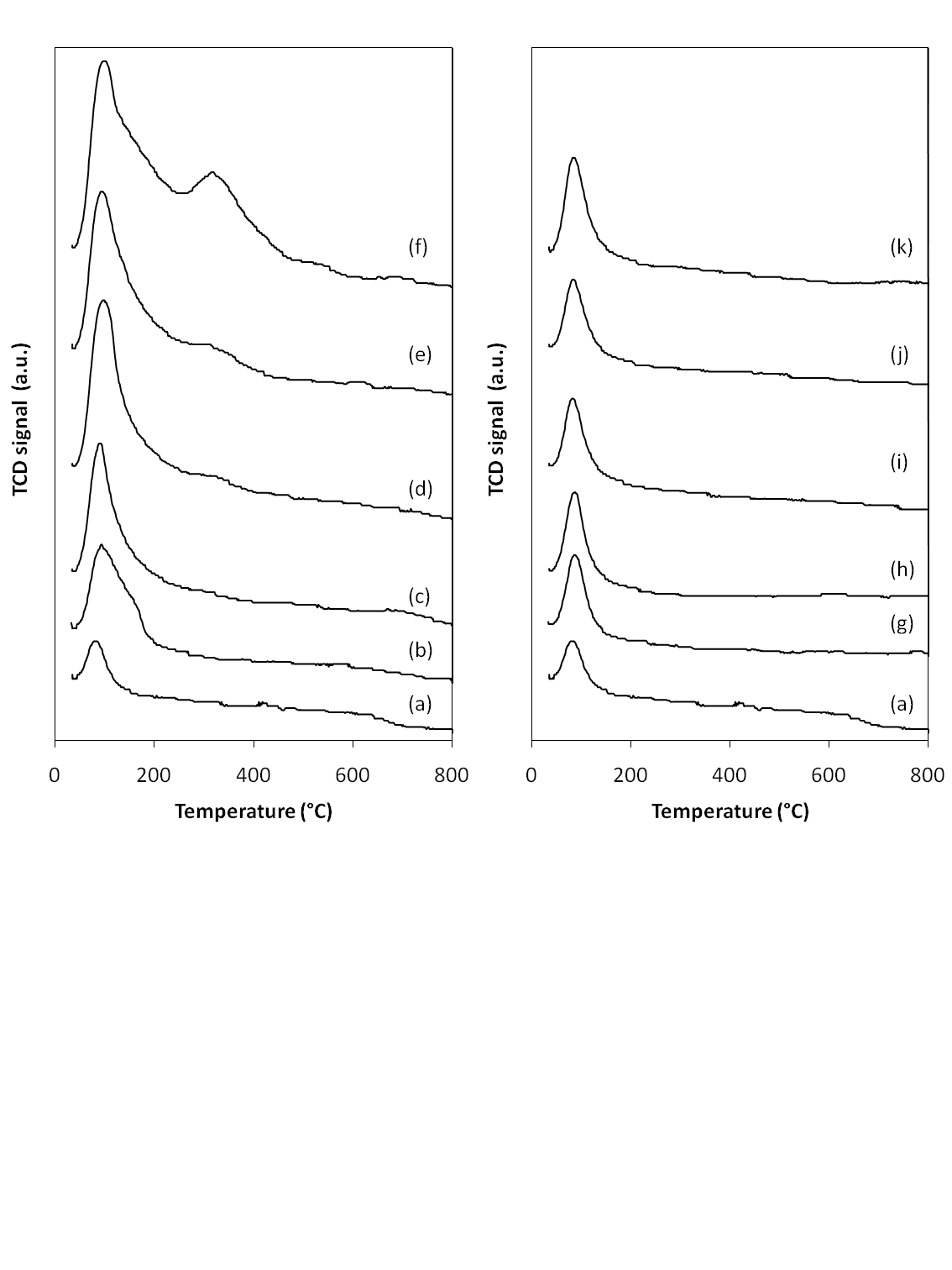


**Figure S5** CO_2_ TPD traces for Mg/γ-Al_2_O_3_ modified supports with different Mg loadings calcined at 550 °C or 800 °C. The traces are: (a) unmodified γ-Al_2_O_3_; (b) 0.5% Mg calcined at 550 °C; (c) 2% Mg calcined at 550 °C; (d) 3% Mg calcined at 550 °C; (e) 4% Mg calcined at 550 °C; (f) 6% Mg calcined at 550 °C; (g) 0.5% Mg calcined at 800 °C; (h) 2% Mg calcined at 800 °C; (i) 3% Mg calcined at 800 °C; (j) 4% Mg calcined at 800 °C; (k) 6% Mg calcined at 800 °C

**Figure S6** Amount of Mg dissolved and not dissolved from Mg/γ-Al_2_O_3_ modified supports calcined after Mg addition at either (a) & (b) 550 °C or (c) & (d) 800 °C washed with either (a) & (c) acetic acid or (b) & (d) ammonium acetate buffer.

**Figure S7** Amount of Al dissolved from Mg/γ-Al_2_O_3_ modified supports calcined after Mg addition at either 550 °C or 800 °C washed with either (a) acetic acid or (b) ammonium acetate buffer. Washed, unmodified γ-Al_2_O_3_ is shown for comparison.

**Figure S8** Temperature of 2^nd^ peak maxima (Co(II)-Co(0)) from HT TGA traces in 5 % H_2_/N_2_ for Co/Ru/Mg/γ-Al_2_O_3_ catalysts, calcined at 550 °C after Mg addition.

**Figure S9** Conversion data with time online for Co/Ru/Mg/γ-Al_2_O_3_ catalysts; (a) 0% Mg (b) AW2; (c) AW3; (d) AW4; (e) AW5 and (f) AW6. GHSV = gas hourly space velocity.

**Figure S10** Selectivity data with time online for Co/Ru/Mg/γ-Al_2_O_3_ catalysts; (a) CH_4_ selectivity and (b) C_5+_ selectivity. Black circles – 0% Mg; Blue filled circles – AW2; Blue open circles – AW3; Blue filled squares – AW4; Red open circles – AW5; Red filled squares – AW6

**Figure S11** Arrhenius plots for samples 0% Mg, AW2 and AW3 from site-time yields calculated at the different testing temperatures

**Table S1 -** Summary of XRF, XRD, TPR and H_2_ chemisorption data for scale-up Co/Ru/Mg/γ-Al_2_O_3_ catalysts prepared with acid washes after Mg addition. Amount of Mg incorporated into Mg_x_Co_3-x_O_4_ spinel was calculated by comparison to literature values reported by Krezhov and Konstantinov^17^.

| Nominal Mg loading (wt%) | Calcination T after Mg addition (°C) |  |  | XRF | | |  | XRD | | |  | TPR | |  | H_2_ Chemisorption | |
| --- | --- | --- | --- | --- | --- | --- | --- | --- | --- | --- | --- | --- | --- | --- | --- | --- |
|  |  | Wash |  | Mg (wt%) | Al (wt%) | Co (wt%) |  | Co_3_O_4_ lattice parameter (Å) | x in Mg_x_ Co_3-x_O_4_ | Co_3_O_4_ PS (nm) |  | Peak pos. (°C) | Peak pos. (°C) |  | CoSA (m^2^/g_Cat_) | CoSA (m^2^/g_Co_) |
| 0 | N/A | AcOH |  | 0.00 | 36.89 | 17.28 |  | 8.0799 (3) | 0.00 | 12.6 |  | 270 | 440 |  | 10.3 | 59.6 |
| 0 | N/A | Buff |  | 0.00 | 36.73 | 17.28 |  | 8.0783 (3) | 0.00 | 13.0 |  | 272 | 453 |  | 10.8 | 62.5 |
| 3 | 550 | AcOH |  | 1.97 | 35.58 | 17.22 |  | 8.0834 (3) | 0.09 | 11.5 |  | 286 | 503 |  | 10.2 | 59.2 |
| 6 | 550 | N/A |  | 5.03 | 32.23 | 17.40 |  | 8.1106 (5) | 0.60 | 12.5 |  | 306 | 573 |  | 10.3 | 59.2 |
| 6 | 550 | AcOH |  | 2.48 | 34.82 | 16.84 |  | 8.0860 (4) | 0.15 | 11.1 |  | 298 | 548 |  | 10.7 | 63.5 |
